# Predictors of surgical site skin infection and clinical outcome at caesarean section in the very severely obese: A retrospective cohort study

**DOI:** 10.1371/journal.pone.0216157

**Published:** 2019-06-27

**Authors:** Michael Dias, Allyn Dick, Rebecca M. Reynolds, Marius Lahti-Pulkkinen, Fiona C. Denison

**Affiliations:** 1 Tommy’s Centre for Maternal and Fetal Health, MRC Centre for Reproductive Health, Queen’s Medical Research Institute, Edinburgh, United Kingdom; 2 Simpson Centre for Reproductive Health, Royal Infirmary, Edinburgh, United Kingdom; 3 British Heart Foundation Centre for Cardiovascular Science, Queen's Medical Research Institute, Edinburgh, United Kingdom; 4 Department of Psychology and Logopedics, Faculty of Medicine, University of Helsinki, Helsinki, Finland; University of Liverpool, UNITED KINGDOM

## Abstract

**Introduction:**

The optimal surgical approach for caesarean section is uncertain in women with very severe obesity (body mass index (BMI) >40kg/m^2^). We aimed to assess maternal and surgical predictors of surgical site skin infection (SSSI) in very severely obese women and to undertake an exploratory evaluation of clinical outcomes in women with a supra-panniculus transverse compared to an infra-panniculus transverse skin incision.

**Material and methods:**

Using a retrospective cohort design, case-records were reviewed of very severely obese women with a singleton pregnancy delivered by caesarean between August 2011 and December 2015 (n = 453) in two maternity hospitals in Scotland. Logistic regression analysis was used to determine predictors for SSSI. Outcomes were compared between women who had a supra-panniculus transverse compared to infra-panniculus transverse skin incision.

**Results:**

Lower maternal age was predictive of SSSI, with current smoking status and longer wound open times being marginally significant. Maternal BMI, suture method and material demonstrated univariate associations with SSSI but were not independent predictors. Women with a supra-panniculus transverse skin incision were older (32.9 (4.4), vs. 30.6 (5.7), p = 0.002), had higher BMI (49.2 (7.1), vs. 43.3 (3.3), p<0.001), shorter gestation at delivery (days) (267.7 (14.9), vs. 274.8 (14.5), p<0.001) and higher prevalence of gestational diabetes mellitus (42.6% vs. 21.9%, p = 0.002). SSSI rates did not differ between supra-panniculus transverse (13/47; 27.7%) and infra-panniculus transverse (90/406; 22.2%; p = 0.395) skin incisions.

**Conclusion:**

SSSI rates are high in very severely obese women following caesarean section, regardless of location of skin incision.

## Introduction

Caesarean section (CS) rates are rising with it being the most common operation undertaken on women of reproductive age. In Scotland, it is estimated 31.1% of singleton births were delivered by CS in 2015/2016, with planned and emergency section rates being 14.3% and 16.9%, respectively [1]. Rates of CS are higher in the obese, with the risk of elective and emergency CS increasing with maternal body mass index (BMI) [2–6].

Maternal obesity is one of the most significant risk factors for surgical site skin infections (SSSI) and wound complications following caesarean delivery [7–13]. Post-operative SSSIs rates range between 3% and 30%, depending on the definition used, length of follow-up and population [7, 9, 11, 13, 14]. SSSI are associated with significant maternal morbidity including increased hospital stay, readmissions, emotional stress, decreased productivity and health care costs [7, 9, 11, 13, 14]. Meta-analyses of randomised controlled trials show that closure of the subcutaneous space [15] [16], administration and timing of antibiotic prophylaxis [17] and method of removing the placenta [18] are important potential predictors of SSSI. However, evidence for other practices to reduce SSSIs including insertion of subcutaneous drains [19] [20], negative pressure dressings [21, 22] and use of barrier retractors [14] is lacking, negative or equivocal with data awaited from ongoing trials.

Very severely obese (BMI>40kg/m^2^) pregnant women are at particularly high risk of SSSI. Normally, a transverse skin incision is made above the symphysis pubis to gain access to the abdomen for caesarean delivery. However, some very severely obese women have a large dependent panniculus which needs to be displaced or retracted before this incision can be performed. These supra-pubic incisions then lie underneath the panniculus in the infra-pannicular skin crease which has a more diverse microbial flora compared to the anterior abdominal wall [23], potentially increasing risk of infection.

Displacement of the panniculus may not be possible in some women due to panniculus size inflammation or ischaemia, or panniculus displacement causing cardio-respiratory compromise. When panniculus retraction is not possible, supra-panniculus vertical skin incisions have been suggested as an alternative surgical approach for caesarean delivery. However, these incisions are associated with increased operative morbidities, including vertical hysterotomy (classical caesarean section), increased blood loss [24] and prolonged post-operative hypoxaemia and respiratory compromise [25, 26]. Evidence is conflicting about whether SSSIs are increased [7, 22, 27, 28] decreased [29] or unchanged [30–33] with this incision.

In non-pregnant patients with a large panniculus, transverse as opposed to vertical supra-panniculus skin incisions are an alternative surgical approach to gaining access to the abdomen. Compared to vertical incisions, transverse incisions are associated with reduced post-operative pain and pulmonary complications [34]. However, evidence is limited about the feasibility, morbidity and outcomes following transverse supra-panniculus skin incisions for caesarean delivery in the very severely obese.

Our study aimed to evaluate the maternal and surgical predictors of SSSI in very severely obese pregnant women undergoing caesarean section, and to undertake an exploratory analysis of clinical outcomes in women with a supra-panniculus transverse compared to infra-panniculus transverse skin incision.

## Methods

### Study population

We performed a retrospective case-note review of all CSs undertaken in very severely obese women with a singleton pregnancy who had a BMI>40kg/m^2^ recorded prior to 20 weeks’ gestation. All CS were undertaken in one of two hospitals in NHS Lothian between August 2010 and December 2015—the Simpson Centre for Reproductive Health at the Royal Infirmary of Edinburgh, which is a tertiary referral centre with more than 6,500 deliveries per annum and St John’s Hospital, Livingston, which is a district general hospital with approximately 2,600 deliveries per annum. Both hospitals use the same electronic maternity record system (maternity TRAK) for clinical records including CS operation notes. To enable a detailed analysis of the surgical and operative predictors of SSSI, women were excluded if the operating room scheduling office system (ORSOS) file associated with the CS or electronic operation note were missing.

In NHS Lothian, all pregnant women with a large panniculus are assessed antentally by a consultant obstetrician to decide the optimal surgical approach for CS. If a supra-pubic skin incision is not possible due to expected difficulties with panniculus retraction, our standard practice has been to use a supra-panniculus transverse as opposed to supra-panniculus vertical skin incision for abdominal entry.

### Data collection

Maternal and offspring data were acquired from maternity TRAK or ORSOS. No patients were involved in the data collection or study design. The data was anonymised at source, with the fields being limited to those collected in the routine clinical dataset.

The following data were collected from the maternal record at booking for all women: maternal age, BMI (kg/m^2^), ethnicity (white, other), parity (P0, P1 or more), smoking status (never, former, current), deprivation quintile (a postcode based Scottish Index of Multiple Deprivation from 2012 with five groups ranging from most deprived index (1) to least deprived index (5) [35] and previous CS (0, 1 or more).

The following variables were collected about delivery: mode of delivery (elective caesarean, emergency caesarean), anaesthesia (epidural, spinal, combined spinal epidural, general anaesthesia), estimated blood loss (mls), admission to maternity high dependency unit (HDU) (yes/no), offspring birthweight (g), macrosomia (>4000g), low birthweight (<2500g), admission to neonatal intensive care (yes/no), sex (male/female), birth outcome (livebirth, stillbirth). In addition, whether a woman had gestational diabetes, (defined as per SIGN Guidelines; [36]) at the time of delivery was recorded.

The following surgical data were collected from maternity TRAK: skin incision type (infra-panniculus incision, defined as a transverse supra-pubic Pfannanstiel or modified Cohen’s approach; or supra-panniculus incision, defined as transverse incision above the pannus allowing transverse opening of aponeurosis and parietal peritoneum), uterine incision (transverse lower segment or vertical hysterotomy), suture material for skin closure (staples, staples with interrupted ethibond, prolene and beads, vicryl, other), method of skin closure (continuous closure, interrupted (either sutures or staples)), subcutaneous fat suture (yes/no), sheath suture (low tensile strength suture, high tensile strength), and subcutaneous drain (yes/no).

The ORSOS files were linked to the maternity TRAK record and used to calculate: operation time (time into anaesthetic room/theatre to time of incision closure), wound open time (time of incision to time of incision closure), total theatre time (time into anaesthetic room to time of leaving theatre).

### Surgical site skin infection

The postnatal maternity TRAK records were interrogated to determine whether a woman did or did not have a SSSI following CS. A SSSI was defined using the NHS Education for Scotland SSSI definition [37] of a superficial incisional SSSI of occurring within 10 days of surgery, involving skin and superficial tissue, and where there is a clinical diagnosis including; purulent drainage (with/without laboratory confirmation), pain/tenderness/redness/heat, or diagnosis of SSSI by a surgeon or attending physician. The diagnosis of a SSSI was made by researcher MD. Any uncertainties were discussed with an independent infection control officer and researcher FD, both of whom were blinded to the initial categorisation, with final categorisation reached by consensus.

### Statistical analysis

Data were analysed using the Statistical Package for the Social Sciences (SPSS) Version 22.0. Descriptive statistics were initially used to synthesise the data via; n (%) and mean (±standard deviation). Prior to the statistical analyses, rank-normalization transformations according to Blom’s formula, [38] [(rank number—3/8) / (sample size + 1/4)], were applied to postnatal hospital stay and BMI and square root transformations to the different operative timings to account for skewness and produce normal distributions. Group differences between the two skin incision site groups in demographic characteristics and clinical outcomes and clinical outcomes following SSSI were examined with Student t-test and χ2- test.

Binary logistic regression analysis was used to determine predictors for SSSI in very severely obese women. First, univariate binary logistic regression was implemented to produce crude odds ratios to examine the demographic/surgical predictors of SSSI. Thereafter, we ran a multivariate binary logistic regression model, including all the demographic and surgical variables, which were statistically significant (p<0.05) or marginally significant (p≤0.10) in the univariate analyses to produce adjusted odds ratios. Prior to regression analysis, all continuous variables were standardized and are expressed in standard deviation units (mean = 0, standard deviation = 1) to facilitate the interpretation of effect sizes.

### Ethical approval

Ethical opinion was sought from the South East Scotland Research Ethics Service. The opinion was that it did not need NHS ethical review (NR/162AB4; 08/02/2016) and the study was classed as a service evaluation/audit.

## Results

### Description of data set

Over the study period, 494 CSs were undertaken in women with very severe obesity. 41 of these were excluded because no operation note was recorded on maternity TRAK and/or no ORSOS file was linked to the delivery. The final dataset for the detailed analysis of the risk factors for SSSI following CS in very severely obese women therefore comprised 453 deliveries.

### Demographic and surgical predictors of surgical site infection

22.7% (103/453) of women with very severe obesity were diagnosed with a SSSI following CS. Maternal and offspring demographics and operative predictors of SSSI in women with very severe obesity are demonstrated in Tables 1 and 2.

**Table 1:** The Demographic predictors of surgical site skin infection in women with Class III obesity. The table shows the odds of getting a surgical site skin infection according to the demographic or perinatal factor in question. Women with no surgical site infection are the referent outcome group, and the referent groups for the independent variables are specified in the table columns.

Demographic	No surgical site skin infection (n = 350)	Surgical site skin infection (n = 103)	OR (95% CI)*	p-value
**Age (years)**^**1**^	31.3 (5.6)	29.2 (5.5)	0.69 (0.55–0.87)	0.001
**Body Mass Index**^**1**^	43.7 (4.3)	44.6 (4.3)	1.31 (1.06–1.64)	0.015
**Ethnicity**^**2**^	(n = 325)	(n = 95)		
** White British/Irish**	280 (86.2)	83 (87.4)	Referent	
** Other**	45 (13.8)	12 (12.6)	0.90 (0.45–1.78)	0.761
**Gestation at Delivery (days)**^**1**^	274.3 (14.3)	273.1 (16.0)	0.93 (0.76–1.14)	0.598
**Smoking Status**^**2**^	(n = 346)	(n = 102)		
** Never Smoked**	204 (59.0)	52 (51.0)	Referent	
** Former Smoker**	94 (27.2)	26 (25.5)	1.09 (0.64–1.84)	0.763
** Current Smoker**	48 (13.9)	24 (23.5)	1.96 (1.10–3.49)	0.022
**Deprivation Quintile**^**2**^	(n = 347)	(n = 103)		
** 5**	47 (13.5)	14 (13.6)	Referent	
** 4**	48 (13.8)	12 (11.7)	0.84 (0.35–2.00)	0.693
** 3**	79 (22.8)	16 (15.5)	0.68 (0.30–1.52)	0.346
** 2**	92 (26.5)	30 (29.1)	1.09 (0.53–2.26)	0.807
** 1**	81 (23.3)	31 (30.1)	1.28 (0.62–2.66)	0.499
**Parity**^**2**^				
** 0**	140 (40.0)	52 (50.5)	1.53 (0.98–2.38)	0.059
** 1+**	210 (60.0)	51 (49.5)	Referent	
**Previous Caesarean Section**^**2**^				
** 0**	182 (52.0)	59 (57.3)	Referent	
** 1+**	168 (48.0)	44 (42.7)	0.81 (0.52–1.26)	0.345
**Diabetes**^**2**^				
** No**	267 (76.3)	77 (74.8)	Referent	
** Yes**	83 (23.7)	26 (25.2)	1.09 (0.65–1.81)	0.750

Data are presented as mean (±SD) ^1^ or n (%)^2^.

*Odds ratios (OR) were calculated using univariate logistic regression analysis after gestation at delivery and BMI were rank-normalized using Blom’s formula transformation. Further, in the logistic regression analyses, all continuous variable were converted to standard deviation units (Mean = 0, Standard Deviation = 1) to facilitate the comparison of effect sizes.

Significance *p*<0.05

**Table 2:** Surgical predictors of surgical site skin infection in women with Class III obesity. The table shows the odds of getting a surgical site skin infection according to the surgical factor in question. Women with no surgical site infection are the referent outcome group, and the referent groups for the independent variables are specified in the table columns.

Surgical predictors	No surgical site skin infection (n = 350)	Surgical site skin infection (n = 103)	OR (95% CI)*	P-value
**Skin incision type**^**2**^				
** Infra-panniculus**	316 (90.3)	90 (87.4)	Referent	
** Supra-panniculus**	34 (9.7)	13 (12.6)	1.34 (0.68–2.65)	0.396
**Category of caesarean**^**2**^				
** Elective**	177 (50.6)	42 (40.8)	Referent	
** Emergency**	173 (49.4)	61 (59.2)	1.49 (0.95–2.32)	0.081
**Form of Anaesthesia**^**2**^	(n = 348)	(n = 102)		
** Spinal**	162 (46.6)	35 (34.3)	Referent	
** Epidural**	84 (24.1)	31 (30.4)	1.71 (0.98–2.96)	0.057
** Combined Spinal Epidural**	72 (20.7)	26 (25.5)	1.67 (0.94–2.98)	0.082
** General Anaesthesia**	30 (8.6)	10 (9.8)	1.54 (0.69–3.45)	0.290
**Operative Timings**^**1**^				
** Anaesthesia to Incision (n = 452)**	0:08 (0:05)	0:09 (0:05)	1.07 (0.86–1.33)	0.529
** Wound Open Time (n = 453)**	0:51 (0:16)	0:55 (0:18)	1.24 (1.00–1.54)	0.050
** Total Operative Time (n = 453)**	1:41 (0:35)	1:45 (0:35)	1.12 (0.90–1.39)	0.295
**Method Skin Closure**^**2**^ **(n = 448)**	(n = 346)	(n = 102)		
** Continuous**	149 (43.1)	34 (33.3)	Referent	
** Interrupted (+/- staples)**	197 (56.9)	68 (66.7)	1.51 (0.95–2.40)	0.080
**Suture Material to close skin**^**2**^ **(n = 445)**	(n = 343)	(n = 102)		
** Staples only**	108 (31.5)	29 (28.4)	Referent	
** Staples + ethibond/other**	88 (25.7)	38 (37.3)	1.61 (0.91–2.81)	0.096
** Prolene and Beads**	101 (29.4)	26 (25.5)	0.96 (0.53–1.74)	0.889
** Vicryl Only**	19 (5.5)	3 (2.9)	0.59 (0.16–2.13)	0.418
** Other**	27 (7.9)	6 (5.9)	0.83 (0.31–2.19)	0.704
**Fat Suture**^**2**^ **(n = 451)**	(n = 349)	(n = 102)		
** No**	144 (41.3)	36 (35.3)	Referent	
** Yes**	205 (58.7)	66 (64.7)	1.29 (0.81–2.04)	0.280
**Rectus Sheath Tensile Suture**^**2**^ **(n = 446)**	(n = 345)	(n = 101)		
** No**	219 (63.5)	53 (52.5)	Referent	
** Yes**	126 (36.5)	48 (47.5)	1.57 (1.01–2.46)	0.047
**Subcutaneous Drain**^**2**^ **(n = 451)**	(n = 349)	(n = 102)		
** No**	336 (96.3)	99 (97.1)	Referent	
** Yes**	13 (3.7)	3 (2.9)	0.78 (0.22–2.80)	0.707

Data are presented as mean (±standard deviation) ^1^ or n (%)^2^.

*Odds ratios (OR) were calculated using univariate logistic regression analysis after operative timings were square-root transformed and converted to standard deviation units (mean = 0, standard deviation = 1).

Younger maternal age, higher BMI, being a current smoker, and having a rectus sheath tensile suture (looped PDS, loop maxon or loop ethibond) were significantly associated with an increased risk of SSSI in univariate logistic regression analysis. Nulliparity, emergency caesarean section, staples + ethibond/other suture type close to skin, and having a longer wound open time were associated with marginally increased odds of SSSI.

Table 3 shows the results of the multivariate logistic regression model, which included all the variables showing significant or marginally significant associations in the univariate models. We found that younger age was the only factor that remained significantly associated with SSSI in the multivariate model. Marginally significant predictors were being a current smoker and having a longer wound open time. BMI became non-significant.

**Table 3:** Multivariate logistic regression model of demographic and surgical predictors of surgical site skin infection in women with very severe obesity (n = 433). The model includes all variables with significant or marginally significant associations in univariate models. The table shows the odds of getting a surgical site skin infection, after adjusting for all the other variables and using women with no surgical site infection as the referent outcome group.

Demographic	Adjusted Odds Ratio (95% CI)	P-value
**Age**	0.72 (0.55–0.93)	0.013
**Body Mass Index**	1.21 (0.92–1.60)	0.173
**Smoking Status**		
** Never Smoked**	Referent	
** Former Smoker**	0.93 (0.52–1.65)	0.799
** Current Smoker**	1.85 (0.99–3.46)	0.055
**Parity**		
** 0**	1.12 (0.65–1.93)	0.671
** 1+**	Referent	
**Category of caesarean**		
** Elective**	Referent	
** Emergency**	1.64 (0.88–3.07)	0.118
**Form of Anaesthesia**		
**Spinal**	Referent	
**Epidural**	1.35 (0.69–2.64)	0.385
**Combined Spinal Epidural**	1.53 (0.80–2.93)	0.203
**General Anaesthesia**	1.22 (0.51–2.96)	0.652
**Operative Timings**		
** Wound Open Time**	1.28 (0.99–1.64)	0.060
**Method Skin Closure**		
** Continuous**	Referent	
** Interrupted (+/- staples)**	3.83 (0.31–47.68)	0.296
**Suture Material to close skin**		
** Staples only**	Referent	
** Staples + ethibond/other**	1.52 (0.79–2.92)	0.206
** Prolene and Beads**	4.11 (0.31–53.99)	0.281
** Vicryl Only**	2.94 (0.17–51.03)	0.460
** Other**	3.35 (0.28–40.38)	0.341
**Rectus Sheath Tensile Suture**		
** No**	Referent	
** Yes**	1.00 (0.57–1.74)	0.997

To account for skewness. Rank-normalization according to Blom’s formula has been applied to body mass index and square root transformation to wound open time. All continuous variables are expressed in standard deviation units.

Regarding the outcomes of SSSI, SSSI was associated with a longer postnatal hospital stay [dd:hh:mm] (mean ± SD, 03:22:42 (2:06:29) vs. 03:08:54 (01:21:29), p = 0.017), readmission to triage (30.1% vs. 11.1%, p<0.001) and postnatal inpatient readmission (19.4% vs. 4.6%, p<0.001). The rate of SSSI did not alter over the time period (p = 0.624; data not shown).

### Site of skin incision and clinical outcomes

Of the 453 women with severe obesity, 406 had a infra-panniculus transverse and 47 had a supra-panniculus transverse skin incision. The demographics for women having infra-panniculus transverse and supra-panniculus transverse skin incisions are demonstrated in Table 4. Women undergoing a supra-panniculus transverse skin incision were older, had higher BMI, shorter gestation at delivery and higher prevalence of gestational diabetes mellitus (Table 4).

**Table 4:** Demographic characteristics of women with a singleton pregnancy delivered by caesarean section by skin site incision.

	Site skin incision		
Demographics	Infra-panniculus transverse (n = 406)	Supra-panniculus transverse (n = 47)	P-value*
**BMI**^**1,**^^**3**^	43.3 (3.3)	49.2 (7.1)	P<0.001
**Age (years)**^**1**^	30.6 (5.7)	32.9 (4.4)	P = 0.002
**Ethnicity**^**2**^			P = 0.681
** White British/Irish**	325 (80.0)	38 (80.9)	
** Other**	50 (12.3)	7 (14.9)	
** Missing**	31 (7.6)	2 (4.3)	
**Gestation at Delivery (days)**^**1**^	274.8 (14.5)	267.7 (14.9)	P<0.001
**Smoking Status**^**2**^			P = 0.133
** Never Smoked**	223 (54.9)	33 (70.2)	
** Former Smoker**	110 (27.1)	10 (21.3)	
** Current Smoker**	68 (16.7)	4 (8.5)	
** Missing**	5 (1.2)	-	
**Deprivation Quintile**^**2,**^^**4**^			
** 5**	54 (13.3)	7 (14.9)	P = 0.756
** 4**	54 (13.3)	6 (12.8)	
** 3**	83 (20.4)	12 (25.5)	
** 2**	109 (26.8)	13 (27.7)	
** 1**	104 (25.6)	8 (17.0)	
** Missing**	2 (0.5)	1 (2.1)	
**Parity**^**2**^			P = 0.774
** 0**	173 (42.6)	19 (40.4)	
** 1+**	233 (57.4)	28 (59.6)	
**Diabetes Status during pregnancy**^**2**^			
** No**	317 (78.1)	27 (57.4)	P = 0.002
** Yes**	89 (21.9)	20 (42.6)	

Data are presented as mean (±SD) ^1^ or n (%)^2^.

^3^Defined as maternal BMI recorded prior to 20 weeks gestation

^4^ The deprivation quintile was calculated using the Scottish Index of Multiple Deprivation (SIMD), 2012, which ranks small ‘datazones’ according to employment, income, health, education, skills, training, geographic access to services, crime and housing. Quintiles are calculated from the list of 6505 ‘datazones’– 1 for the most deprived and 5 for the least deprived.

*Significance between maternal/neonatal outcomes and surgical site skin infection is assessed via independent two-tailed t-tests for continuous variables and Chi Squared tests for categorical variables. Significance *p*<0.05. Participants with missing values are excluded from the statistical analyses concerning the variable in question

Table 5 shows the surgical variables, which were associated with supra-panniculus transverse skin incision. These included; elective CS, combined spinal-epidural anaesthesia, longer anaesthesia to incision time, longer wound open time, longer total operative time, interrupted suture technique (±staples) use of staples with ethibond/other, use of subcutaneous fat sutures and the use of a tensile suture for the rectus sheath (Table 5).

**Table 5:** Surgical variables categorised by site of skin incision.

	Skin Incision Type		
Surgical Variable	Infra-panniculus transverse (n = 406)	Supra-panniculus transverse (n = 47)	p-value*
**Mode of Delivery**^**2**^			
** Elective Caesarean Section**	187 (46.1)	32 (68.1)	P = 0.004
** Emergency Caesarean Section**	219 (53.9)	15 (31.9)	
**Form of Anaesthesia**^**2**^			
** Spinal**	187 (46.1)	10 (21.3)	P<0.001
** Epidural**	110 (27.1)	5 (10.6)	
** Combined Spinal Epidural**	72 (17.7)	26 (55.3)	
** General Anaesthesia**	36 (8.9)	4 (8.5)	
** Missing**	1 (0.2%)	2(4.3)	
**Operative Timings (hh:mm)**^**1**^			
** Anaesthesia to Incision**	0:08 (0:05)	0:13 (0:07)	P<0.001
** Wound Open Time**	0:51 (0:16)	1:04 (0:17)	P<0.001
** Total Operative Time**	1:37 (0:31)	2:23 (0.41)	P<0.001
**Suture Type**^**2**^			
** Continuous**	180 (44.3)	3 (6.4)	P<0.001
** Interrupted (+-staples)**	221 (54.4)	44 (93.6)	
** Missing**	5 (1.2)	-	
**Suture Material**^**2**^			
** Staples only**	129 (31.8)	8 (17.0)	P<0.001
** Staples and ethibond/other**	91 (22.9)	35 (74.5)	
** Prolene and Beads**	124 (30.5)	3 (6.4)	
** Vicryl only**	22 (5.4)	-	
** Other**	32 (7.9)	1 (2.1)	
** Missing**	8 (2.0)	-	
**Fat Suture**^**2**^			
** No**	175 (43.1)	5 (10.6)	P<0.001
** Yes**	229 (56.4)	42 (89.4)	
** Missing**	2 (0.5)	-	
**Rectus Sheath Tensile Strength Suture**^**2**^			
** No**	264 (65.0)	8 (17.0)	P<0.001
** Yes**	135 (33.3)	39 (83.0)	
** Missing**	7 (2.7)	-	
**Subcutaneous Drain**^**2**^			
** No**	389 (95.8)	46 (97.9)	P = 0.578
** Yes**	15 (3.7)	1 (2.1)	
** Missing**	2 (0.5)	-	

Data are presented as mean (±SD) ^1^ or n (%) ^2^.

*The significance of the associations between maternal/neonatal outcomes and surgical site skin infection is assessed via independent two-tailed t-tests for continuous and Chi squared tests for categorical variables. Participants with missing values are excluded from the statistical analyses on the variable in question.

Maternal and offspring outcomes categorised by site of skin incision are demonstrated in Table 6. All babies were delivered via a transverse lower segment uterine hysterotomy, regardless of skin incision. Supra-panniculus transverse skin incisions were associated with; longer postnatal hospital stay, greater estimated blood loss, greater HDU admission, readmission to triage, and readmission as an inpatient. The majority of admissions to HDU, triage and inpatient readmissions were due to comorbidities related to maternal obesity such as diabetes or respiratory compromise due to extreme obesity. No woman was admitted to maternity HDU due to wound complications and there was no difference in rates of readmission due to wound problems between groups (data not shown). SSSI rates did not differ between supra-panniculus transverse and infra-panniculus transverse skin incisions (Tables 2 and 6).

**Table 6:** Maternal and offspring outcomes categorised by site of skin incision.

Skin Incision Type			
Outcomes	Infra-panniculus transverse (n = 406)	Supra-panniculus transverse (n = 47)	p-value*
**Maternal Outcome**			
**Wound infection**^**1**^			
**Yes**	316 (77.8)	34 (72.3)	P = 0.395
**No**	90 (22.2)	13 (27.7)	
**Postnatal Hospital Stay (DD/HH/MM)**^**2**^	03:10:49 (2:10:19)	03:22:28 (01:19:54)	P = 0.018
**Lochia Status**^**1**^			
**Minimal**	233 (57.4)	25 (53.2)	P = 0.739
**Moderate**	165 (40.6)	21 (44.7)	
**Severe**	4 (1.0)	-	
**Offensive**	4 (1.0)	1 (2.1)	
**Estimated Blood Loss (ml)**^**2**^	705.1 (424.5)	1044.5 (744.8)	P = 0.004
**Maternal High Dependency Unity Admission (postnatal)**^**1**^			
**No**	364 (89.7)	31 (66.0)	P<0.001
**Yes**	42 (10.3)	16 (34.0)	
**Readmission**^**1**^			
**None**	318 (78.3)	29 (61.7)	P = 0.029
**Triage**	57 (14.0)	13 (27.7)	
**Inpatient**	31 (7.6)	5 (10.6)	
**Offspring Outcome**			
**Birth Weight (g)**^**2**^	3576.5 (678.1)	3594.2 (757.7)	P = 0.867
**Neonatal Intensive Care Unit Admission**			
**No**	362 (89.2)	38 (80.9)	P = 0.089
**<24 Hours**	11 (2.7)	4 (8.5)	
**>24 Hours**	27 (6.7)	4 (8.5)	
**Missing**	6 (1.5)	1 (2.1)	
**Sex**^**1**^			
**Female**	191 (47.0)	23 (48.9)	P = 0.806
**Male**	215 (53.0)	24 (51.1)	

Data are presented as mean (±SD) ^1^ or n (%)^2^.

*Significance between maternal/neonatal outcomes and surgical site skin infection is assessed via independent two-tailed t-tests for continuous variables and Chi Squared for categorical variables.

Significance p<0.05. Participants with missing values are excluded from the statistical analyses for

the variable in question.

## Discussion

### Main findings

In this retrospective case-note review, we demonstrate that lower maternal age and were predictive of SSSI, with current smoking status and longer wound open times being marginally significant. Maternal BMI, suture method and material demonstrated a univariate association with SSSI but were not independent predictors in women with severe obesity. In an exploratory analysis, compared to infra-panniculus transverse skin incisions, maternal intra- and post-operative morbidity was increased following a supra-panniculus transverse skin incision and there was no difference in SSSI rates.

### Validity of results

Severe obesity significantly increases risk of SSSI following CS [7–13], and with almost one quarter of women with severe obesity suffering SSSI. We demonstrate that smoking and increased wound open times are associated with increased risk of SSSI in the very severely obese. Both are factors modifiable and have potential to reduce the risk of SSSI. We also identified that younger age is an independent risk factor for SSSI. Targeting younger women who smoke with specific information about post-operative wound hygiene may reduce infection rates. Surprisingly, unlike other studies we failed to correlate SSSI rates with subcutaneous drain use [7], suture material [39, 40], subcutaneous fat sutures [41] or total operative time [42] which could be due to a smaller sample size, routine use of antibiotic administration prior to skin incision and other confounding factors which we were unable to correct for.

Performing CSs in women with very severe obesity presents numerous operative and anaesthetic difficulties [5, 10, 11, 32, 43–45]. Increasingly, alternative surgical approaches are being advocated in attempt to reduce complications and circumvent the operative challenges of operating on the very severely obese. In a large retrospective cohort study (n = 3200 women with severe obesity in 19 centres) by Marrs et al [29], wound complication rates following CS were lower in women who had a supra-panniculus vertical incision compared to infra-panniculus transverse incision after adjustment for confounders on multivariate regression analysis [29]. In our smaller study, we were unable to replicate these findings with there being no statistically significant difference in SSSI rates between women with a supra-panniculus transverse incision compared to those with an infra-panniculus transverse incision. However, maternal BMI, rates of diabetes and operative timings, which are all independent predictors of SSSI [7, 13, 31, 32, 46, 47], were all higher/longer in women with a supra-panniculus transverse skin incisions. It is possible that these factors could be concealing superiority for the supra-panniculus transverse approach in terms of SSSI which we were unable to identify due to the low numbers of women with this approach which limited the analysis.

All women who had supra-panniculus transverse skin incisions, had their baby delivered via a transverse lower uterine segment hysterotomy. Compared to a classical vertical hysterotomy, which is often required when a vertical supra-panniculus incision is used for abdominal entry, this uterine incision has less operative morbidity and a much lower incidence of uterine rupture in a subsequent pregnancy [48]. It may therefore be attractive to consider a transverse supra-pannicular incision when an infra-panniculus supra-pubic incision is not possible due to inability to retract a large panniculus. However, this decision needs be balanced against our findings that women with a supra-panniculus transverse incision were more likely to be admitted to the maternal HDU, have a higher blood loss at delivery, to attend triage postnatally and be readmitted than women who had an infra-panniculus skin incision. For most women, admission to HDU or postnatal readmission was due to obesity related co-morbidities such as maternal diabetes and post-operative respiratory compromise due to maternal BMI. The mean BMI in women with supra-panniculus incisions was higher than those with infra-panniculus incisions and this may have influenced location of post-operative care. However, we accept that our numbers are too small to definitively conclude that site of skin incision did not additionally contribute to clinician decision-making when choosing the most appropriate location for immediate post-operative care.

### Strengths and limitations

A strength is that, by using detailed routinely collected data, we were able to examine the effect of wide range of demographic and surgical variables on SSSI. Our results are therefore relevant to clinical practice where different surgical techniques are used. We used a robust definition of SSSI, as endorsed by “NHS Education for Scotland” [37] and each case was independently checked and verified by two investigators, blinded to the other’s categorisation.

We accept the retrospective case-note design is a study limitation with our sample size and analysis being limited by the study population, data fields available and quality of data recorded. For the majority of the outcomes, we had a relatively low proportion of missing data. However, we accept that some important predictors of SSI’s, such as timing of antibiotic administration and method of placenta removal [18], because this information is routinely recorded in the paper and not the electronic clinical records which were used for analysis. Thus, although it is routine practice to administer prophylactic antibiotics to women undergoing CS in NHS Lothian to reduce risk of SSI, we were not able to include antibiotic administration and timing or method of placenta removal in our modelling. Although we did not observe any systematic change in operative practices over the time period studied, we accept that this may have been an additional confounder, which we were unable to adjust for. Similarly, we do not have information about the level of experience and grade of operator undertaking the CS, which may have confounded the results. A lack of a defined protocol for skin incision choices also adds subjectivity to the choice that individual clinicians made when choosing which skin incision to use.

## Conclusion

We have demonstrated that SSSIs are common in women with severe obesity, and have identified some potentially modifiable risk factors. In an exploratory analysis, we also demonstrate that although a lower segment caesarean section was possible in every case of supra-panniculus transverse incisions, maternal intra- and post-operative morbidity was increased and there was no difference in SSSI rates compared to supra-panniculus transverse incisions. Given the high burden of post-operative morbidity, rising levels of maternal obesity, high CS rates and multifactorial nature of SSSIs, our study highlights the need for further research to reduce the burden of morbidity in women with Class III obesity undergoing caesarean delivery and patient experience in relation to different surgical approaches.
